# Progressive joint limitations as the first alarming signs in a boy with short – limbed dwarfism: A case report

**DOI:** 10.1186/1757-1626-1-112

**Published:** 2008-08-19

**Authors:** Ali Al Kaissi, Klaus Klaushofer, Franz Grill

**Affiliations:** 1 Ludwig-Boltzmann Institute of Osteology at the Hanusch Hospital of WGKK and AUVA Trauma Centre Meidling, 4th Medical Department, Hanusch Hospital, Vienna, Austria; 2 Orthopaedic Hospital of Speising, Paediatric Department, Vienna, Austria

## Abstract

**Introduction:**

Contracture is a condition of abnormal shortening or shrinkage of a muscle, and or a tendon often with persistent flexion or distortion at a joint. Careful documentation of the kind of contractures encountered in different paediatric disorders is important in distinguishing a specific subtype. Achondroplasia has been considered as the most common short-limbed dwarfism syndrome, but there are a variety of other syndromes within this category, and other types of limb shortening.

**Case presentation:**

We report on a 5-year-old boy of Austrian origin who manifests progressive joint limitations in connection with a dysplastic form of short-limbed dwarfism namely chondrodysplasia punctata-tibial-metacarpal-type. Progressive joint limitations of maximal intensity over the hip, and the ankle joints were the main presenting features.

**Conclusion:**

Osteochondrodysplasias involve abnormal bone or cartilage growth leading to skeletal maldevelopment, often short-limbed dwarfism. Diagnosis is by physical examination, radiographic documentation, and, in some cases, genetic testing. In patients with chondrodysplasia punctata, early life radiographic examination is fundamental, since resolution of the punctate calcifications leaving abnormal epiphyses and flared and irregular metaphyses after age one to three years seems to be characteristic.

## Background

The term dwarf is somewhat ambiguous, but generally refers to a condition with shortening of a limb or the spine such that the pattern is recognized as a syndrome and that the affected region is generally shorter than 97% of children of the same age. It should be noted, however, that children with very short stature but no limb shortening may simply be normal or may have a metabolic condition such as malnutrition. There are several short limb/skeletal dysplasias that can be associated with variable degrees of joint limitations. Chondrodysplasia punctata in general is a very rare, little-understood disorder in which spots of opaque calcifications are observed in the epiphyseal cartilage at birth. Many infants die within the first year; those who live may exhibit dwarfism, mental retardation, and variable skeletal and extraskeletal malformation complex. In the present case the diagnosis of chondrodysplasia punctata-tibial-metacarpal-type was made at the age of 2 years through the well-defined punctate calcifications in the epiphyseal cartilage.

## Case presentation

A 5-year-old boy of Austrian origin was referred to the orthopaedic department because of short-limbed-dwarfism associated with significant progressive joint mobility contractures. He was the product of uneventful gestation. At birth his weight, length and head circumference were below the 3 rd centile. No abnormality was detected at physical examination, apart from prenatal dwarfism. The parents were unrelated and were clinically normal. Family history was negative for heritable diseases such as metabolic disorders or other neurological diseases. No other family members were known to have similar phenotype.

Motor development was normal, but recently out toeing with significant restrictions over the hip and ankle joints were the main complaints. The child is hyperactive with attention deficit disorder. He had a history of chronic otitis media.

**Figure 1:**
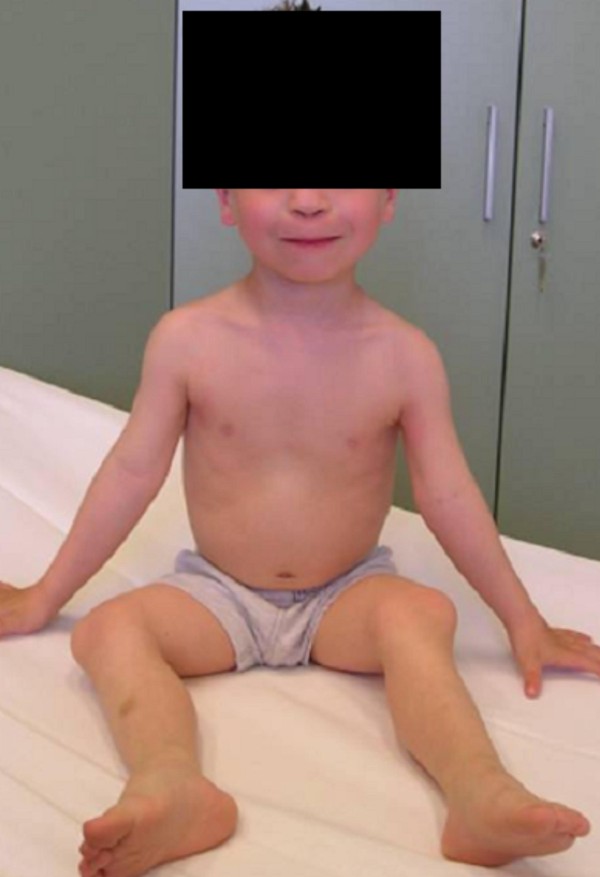
Facial features showed mid-face hypoplasia, upward slant of the palpebral fissures, a small/flat nose with depressed nasal bridge, a long upper lip with a flat-long philtrum, macrostomia and a high palate and a thin vermilion border.

**Figure 2:**
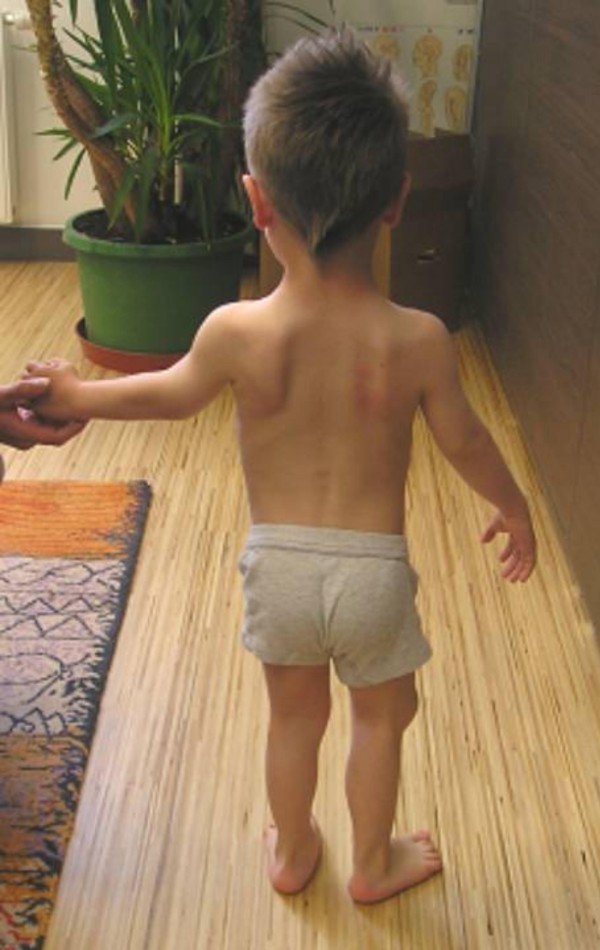
Short-limbed dwarfism was notable with out-toeing and a unilateral dislocated patella.

Examination at the age of 5 years showed short stature of -4SD. Facial features showed mid-face hypoplasia, upward slant of the palpebral fissures, a small/flat nose with depressed nasal bridge, a long upper lip with a flat-long philtrum, macrostomia and a high palate and a thin vermilion border (figure 1). Short-limbed dwarfism associated with out-toeing and a unilateral dislocated patella was evident (figure 2). Vision was normal, but intelligence was evidently subnormal. Speech delay was notable because of chronic secretary otitis media.

There was stiffness over his spine associated with loss of the physiological lumbar lordosis. Pain over the hip and ankle joints was of recent onset. Pain over the posterior aspect of his thighs and groin was present as well. Examination of the hips showed a restricted painful range of the hip bilaterally (this was maximally through abduction). Similarly restricted elbow joint mobility because of a hypoplastic/dislocated ulna. Unilateral patellar dislocation was present. Out-toeing was additional feature associated with significant stiffness over the ankle joints. The spine was stiff associated with loss of the physiological lumbar lordosis. Basic biochemical defect has not been identified. Abdominal ultrasound did not reveal associated abnormalities. Chromosomal analysis for the child and his parents showed no abnormalities. Besides, hormonal investigations included thyroid hormones; adrenocorticotropic hormone and growth hormone were negative as well. Echocardiodoppler showed sinus dysrhythmia with no obvious cardiac lesion. Abdominal ultrasound did not reveal associated abnormalities.

### Radiographic examination

**Figure 3:**
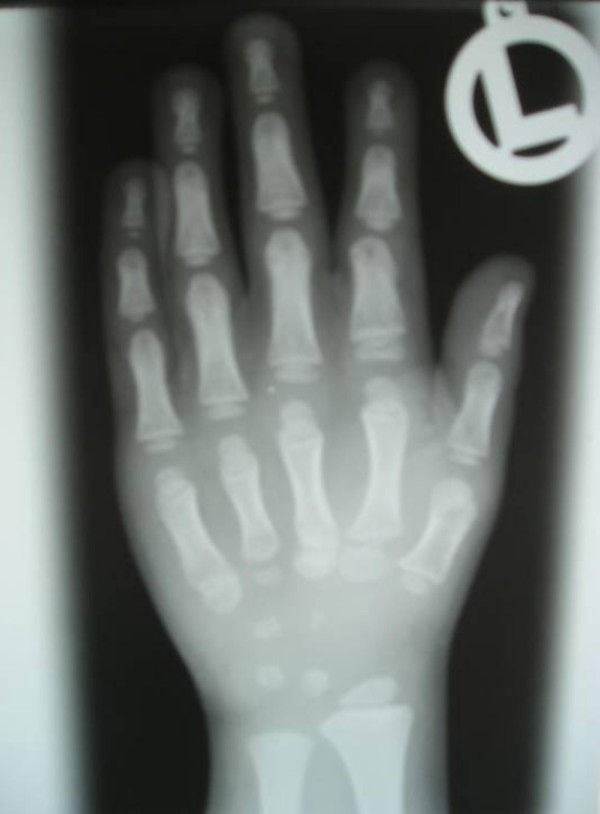
Anteroposterior hand radiograph showed defective ossification of the carpal bones, the proximal phalanges of the first and fourth fingers were short and broad.

**Figure 4:**
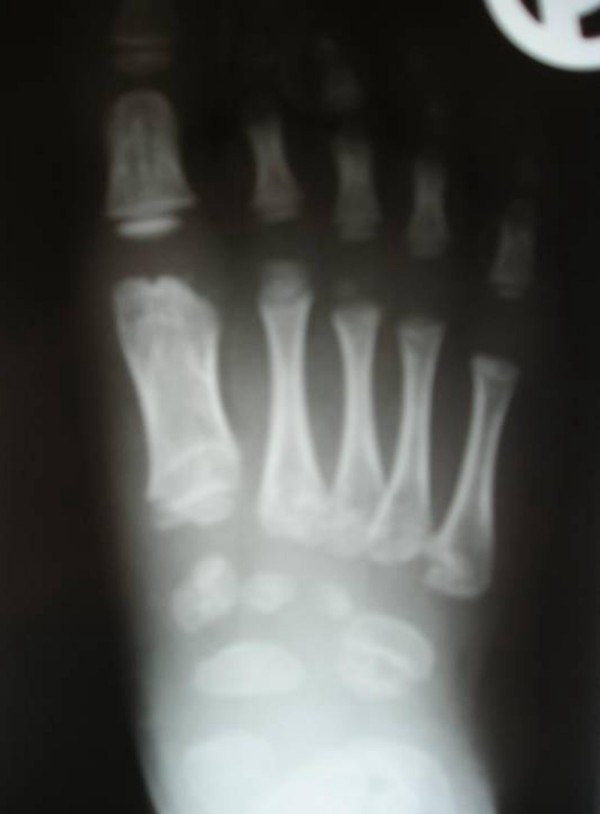
**Anteroposterior foot radiograph showed defective ossification of the tarsal bones as well as brachymetatarsia and Brachyphalangia were present.** There was epiphyseal clefting of the distal first and the proximal 5th metatarsophalangeal joints.

**Figure 5:**
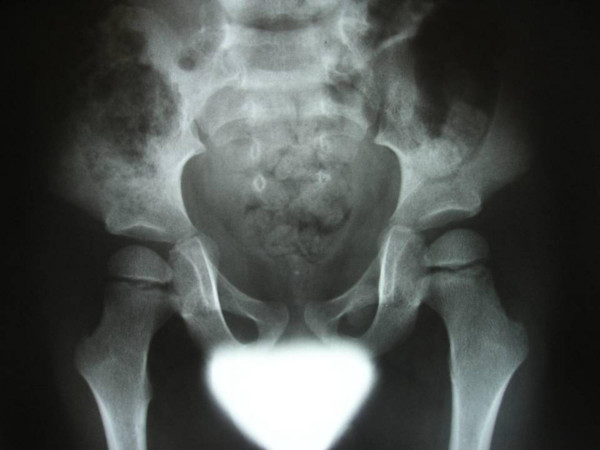
Anteroposterior lower pelvis and long bones radiograph showed Coxa valga, long femoral neck and inward curvation of the femoral shafts associated with widened metaphyses of the inferior femora.

**Figure 6:**
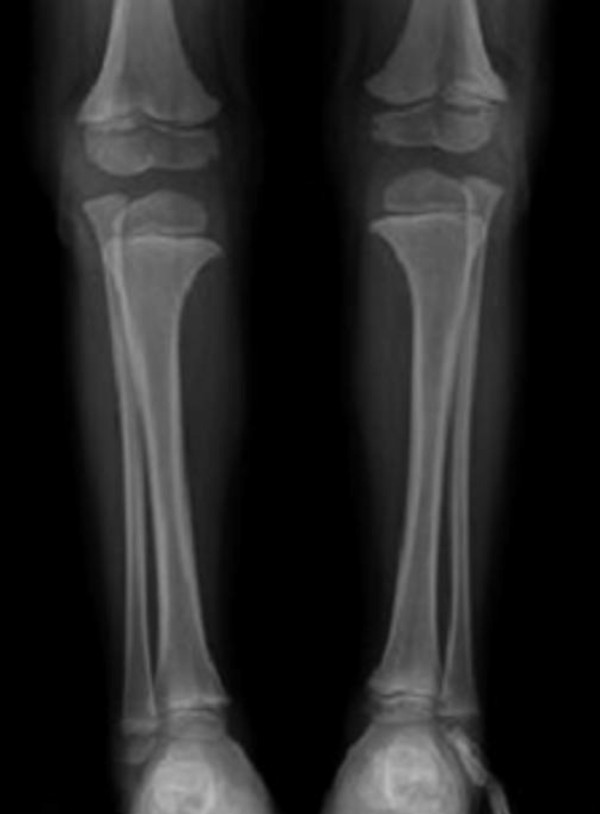
**Anteroposterior radiograph of the lower limbs showed unduly long fibulae causing effectively medial tibial torsion and a tongue like projection of the medial metaphyses of the proximal tibia.** Significant bilateral tibial dysplasia was apparent.

**Figure 7:**
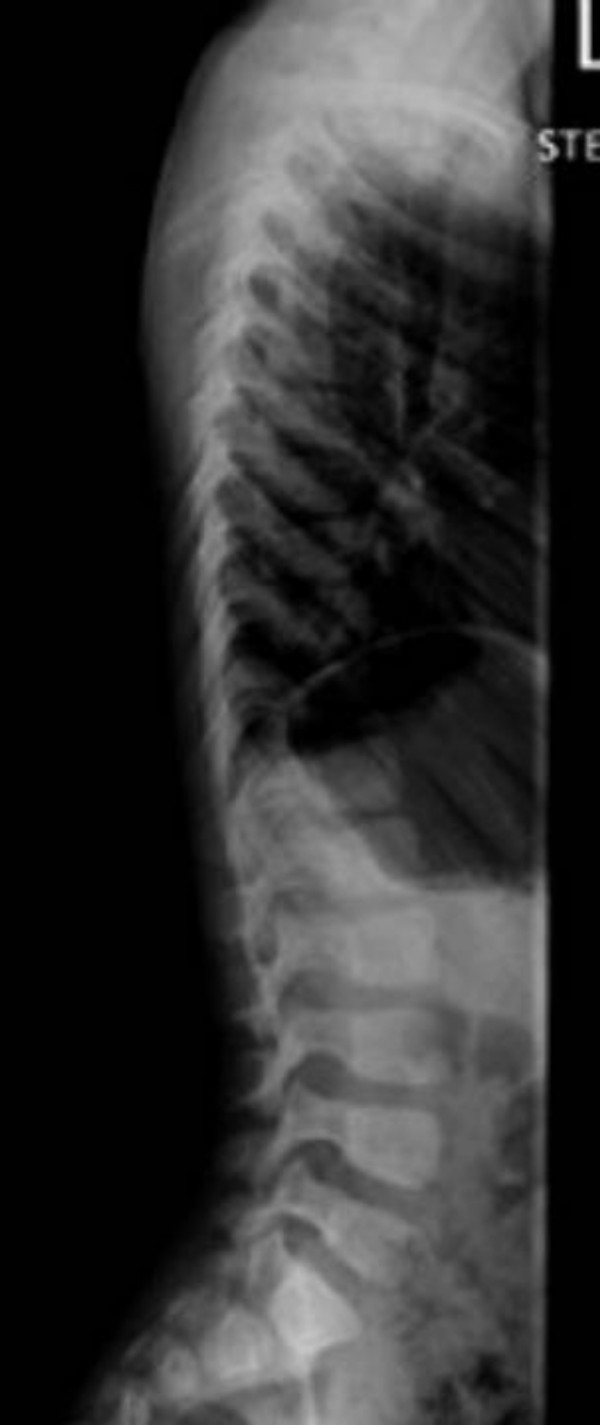
Lateral spine radiograph showed normal vertebral anatomy but with loss of the physiological lumbar lordosis.

Anteroposterior hand radiograph showed defective ossification of the carpal bones and short tubular bones. The proximal phalanges of the first and fourth fingers were short and broad (figure 3). Anteroposterior foot radiograph showed defective ossification of the tarsal bones as well as brachymetatarsia and brachyphalangia were present. There was epiphyseal clefting/fragmentation of the first distal and the proximal 5th metatarsophalangeal joints (figure 4). Anteroposterior lower pelvis and long bones radiograph showed Coxa valga, long femoral neck and inward curvation of the femoral shafts associated with widened metaphyses of the inferior femora (figure 5). Anteroposterior radiograph of the lower limbs showed unduly long fibulae causing effectively medial tibial torsion and a tongue like projection of the medial metaphyses of the proximal tibia and demineralisation. Significant bilateral tibial dysplasia was apparent (figure 6). Lateral spine radiograph showed normal vertebral anatomy but with loss of the physiological lumbar lordosis (figure 7).

Treatment was primarily based upon avoidance of weight bearing and lessening from sport activities. Physical therapy and hydrotherapy have been indicated to maintain the hips range of movement.

## Discussion

Joint contractures are not uncommon impairments occur in association with a number of syndromic associations such as Chondrodysplasia punctata, Geleophysic dysplasia, Freeman-Sheldon syndrome, Stüve-Wiedemann syndrome and Schwartz-Jampel syndrome. Selected progressive neuromuscular disease conditions can also lead to such increased disabilities as worsening motor performance, decreased mobility, loss of skills and sometimes pain. [1-7].

Chondrodysplasia punctata is erratic cartilage calcification during growth, which produces the heterogeneous group of disorders that results in small ossification centers in the epiphyseal cartilage of the long bones and spine, skin lesions, cataracts, craniofacial dysmorphism, joint contractures, and cardiac malformation. Chondrodysplasia punctata is also known as chondrodystrophia calcificans congenita or congenital stippled epiphyses. The disease variably defined as mesomelic or rhizomelic dwarfism depending on the gene transmission. In surviving children, abnormal growth leads to dysmorphism, kyphoscoliosis, limb shortness, and luxation of the hip and progressive joint limitations [1,8,9].

Craniofacially, a combination of dysmorphic features might be encountered in children with chondrodysplasia punctata such as asymmetric head, frontal bossing, flat nasal bridge, dysplastic auricles, mongoloid palpebral fissures, hypertelorism, and high arched palate. Skeletal abnormalities, however such as asymmetric mild shortening of all long bones, bowing, stippled epiphysis, vertebral scoliosis, clefting, or wedging, flexion Contracture of the joints, clubfoot or valgus deformity are frequent features [10,11].

Rittler et al., [1] described seven unrelated infants with a relatively mild form of chondrodysplasia punctata. Short-limbed dwarfism, small hands and feet were evident at birth. Shortening of the tibiae and 4th metacarpals was particularly characteristic.

Matsui et al., [12] reported a further male case. A long-term follow-up of patients reported by Savarirayan et al., [13] in which, intelligence was normal and all 3 were active. The adult heights ranged from 138 cm to 148 cm. All had recurrent patellar dislocations, one had spinal stenosis and one had had a hip replacement. Wester et al., [14] reported a two-year-old boy with features of tibia-metacarpal type chondrodysplasia punctata whose mother took phenytoin throughout the pregnancy. To classify the different forms of chondrodysplasia punctata has been difficult [15]. Gene mapping has confirmed the heterogeneity of the various types. Limited movement in the joints in children with skeletal dysplasias requires particular attention and management. The progressive limitations in joints mobility in our patient were further clarified after age of 4 years. During his infancy punctate calcification in the tibiae, metacarpal, calcaneus, and metatarsal were evident. Growth and developmental progress was slightly improved, but the final phenotype included short-limbed dwarfism, progressive joint limitations and subnormal intelligence with persistence of the typical facies were characteristic. In our present patient inheritance appear to be sporadic.

## Conclusion

A better knowledge of the etiology, genetics, pathogenesis, and natural history in patients with osteochondrodysplasias is the corner stone for a comprehensive management. Early recognition of affected children should allow for aggressive malformation complex control and expectant management of multiple associated problems.

## Abbreviations

SD: Standard deviation.

## Competing interests

The authors declare that they have no competing interests.

## Authors' contributions

All of the authors were involved in the clinico-radiographic assessment and finalising the paper. All authors have read and approved the final version of the paper.

## Consent

Written informed consent was obtained from the parents for the purpose of publication of the manuscript and figures of their child. A copy of the written consent is available for review by the editor-in-Chief of this journal.
